# Circadian Rhythm Influences the Promoting Role of Pulsed Electromagnetic Fields on Sciatic Nerve Regeneration in Rats

**DOI:** 10.3389/fneur.2017.00101

**Published:** 2017-03-15

**Authors:** Shu Zhu, Jun Ge, Zhongyang Liu, Liang Liu, Da Jing, Mingzi Ran, Meng Wang, Liangliang Huang, Yafeng Yang, Jinghui Huang, Zhuojing Luo

**Affiliations:** ^1^Institute of Orthopaedics, Xijing Hospital, The Fourth Military Medical University, Xi’an, China; ^2^Department of Orthopaedics, 323rd Hospital of PLA, Xi’an, China; ^3^Department of Anatomy, The Fourth Military Medical University, Xi’an, China; ^4^Department of Orthopaedics, 161st Hospital of PLA, Wuhan, China; ^5^Faculty of Biomedical Engineering, Fourth Military Medical University, Xi’an, China; ^6^Department of Anesthesiology, Xijing Hospital, The Fourth Military Medical University, Xi’an, China; ^7^General Political Department Hospital of PLA, Beijing, China

**Keywords:** pulsed electromagnetic fields, circadian rhythm, chronotherapy, peripheral nerves, nerve regeneration, functional recovery

## Abstract

Circadian rhythm (CR) plays a critical role in the treatment of several diseases. However, the role of CR in the treatment of peripheral nerve defects has not been studied. It is also known that the pulsed electromagnetic fields (PEMF) can provide a beneficial microenvironment to quicken the process of nerve regeneration and to enhance the quality of reconstruction. In this study, we evaluate the impact of CR on the promoting effect of PEMF on peripheral nerve regeneration in rats. We used the self-made “collagen-chitosan” nerve conduits to bridge the 15-mm nerve gaps in Sprague-Dawley rats. Our results show that PEMF stimulation at daytime (DPEMF) has most effective outcome on nerve regeneration and rats with DPEMF treatment achieve quickly functional recovery after 12 weeks. These findings indicate that CR is an important factor that determines the promoting effect of PEMF on peripheral nerve regeneration. PEMF exposure in the daytime enhances the functional recovery of rats. Our study provides a helpful guideline for the effective use of PEMF mediations experimentally and clinically.

## Introduction

Peripheral nervous injury (PNI), as a common disease, has very high incidence around the world (1), which often leads to the absence of sensory function and disability of motor function. Currently, peripheral nerve regeneration is still a challenge in the field of regenerative medicine. Clinically, an ideal repair should achieve wound healing without cicatrization and mismatch to fast functional recovery (2). The direct end-to-end nerve suture can be used because of the natural re-growth of small gap injuries (1–2 mm). However, for longer gap of nerve defects, it needs a graft to provide a bridge for regenerating axons. At present, autologous nerve graft has been widely used for bridging long nerve defects (3). However, autograft transplantation is still suffered from additional drawbacks, such as the limitation of donor nerve availability and postoperative complications of donor sites (4–6). In recent years, many synthetic tubular nerve scaffolds have been developed to bridge long nerve defect (7, 8). Synthetic tubular nerve scaffolds provide an air tight microenvironment to guide the regenerating axons so that they show the potential to restore nerve regeneration. Performance of the nerve scaffolds can be optimized by porosity (9), surface roughness (10), and electrical properties (11, 12). Although collagen-chitosan nerve scaffolds can achieve axonal regeneration when bridging over 10 mm nerve gaps in rats (13–15), the microenvironment at the local site of scaffolds still needs to be optimized to further enhance axonal regeneration and functional recovery.

Pulsed electromagnetic fields (PEMF), as an effective non-invasive method, have shown the potential to improve peripheral nerve regeneration since 1980s (16, 17). PEMF can not only promote the survival and neuronal differentiation of cells (18) but also control the migration orientation of Schwann cells (SCs) as well as the growth direction of regenerating axons (19–21). The stimulation efficacy of PEMF is influenced by many known factors such as the ubieties between PEMF generator and subjects (22), PEMF parameters (23), and exposure duration (24). However, as PEMF radiation is a complex process, and additional factors that affect the efficacy of PEMF treatment in peripheral nerve regeneration still remain to be determined.

Circadian rhythm (CR), as an acritical and common factor, closely links with mammals’ behaviors and physiological changes (25). It makes the metabolism more reasonable and the energy conversion more effective (26, 27). From the concept of chronotherapy that was first put forward since 1960s (28), the terms “chronopharmacology” and “chronotherapy” become increasingly popular. As the CR is the core of chronotherapy, numerous studies showed that CR affects treatment of many diseases, such as cancers (29, 30), bronchial asthma (31–33), and cardiovascular diseases (34–36). For the treatment of hypertension, there have been four drug delivery strategies of chronotherapy (28, 37, 38). In addition, CR has been reported to influence the prevention of osteoporosis by PEMF (39). However, whether the CR can influence the efficacy of PEMF stimulation in peripheral nerve regeneration has never been studied. Therefore, the present study is to investigate the effect of CR on the efficacy of PEMF in the treatment of PNI in a rat model.

## Materials and Methods

### Fabrication of the Chitosan-Collagen Conduits and Microstructure Observation

**Figure 1:**
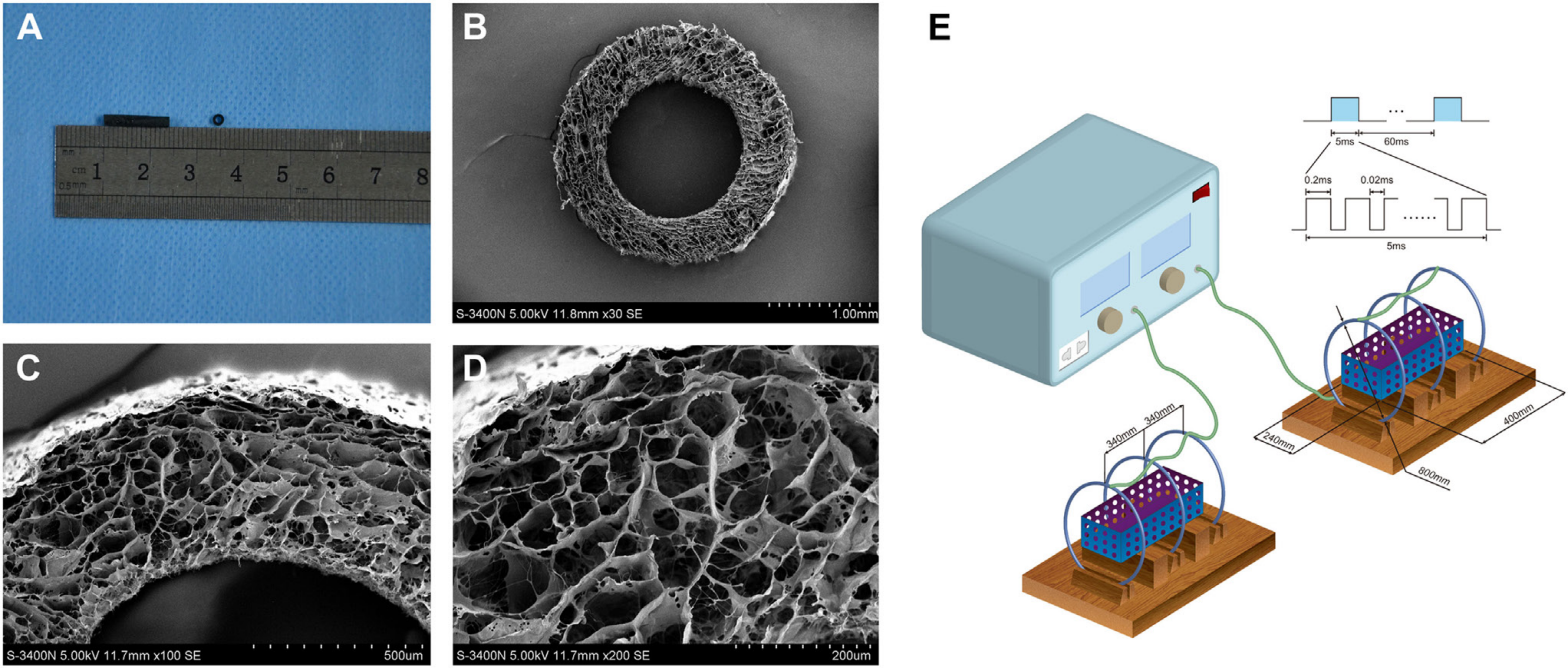
**The general structure (A) and microstructural appearance of chitosan-collagen conduit under scanning electron microscopy (B–D) were shown**. The schematic illustration of the device used to expose experimental animals to pulsed electromagnetic fields (PEMF) was shown **(E)**. It included in a PEMF generator and several identical Helmholtz coils with 800 mm coil diameters. The coils were wired to a pulse generator, which produced an electromagnetic field signal with a frequency of 2 Hz, produced the magnetic field with amplitude of 0.3 mT, and a pulse duration of 20 ms and placed with interval of 304 mm in the same axis. Each coil was composed by enameled coated copper wire with 0.8-mm diameter. This ubiety of three coils reduced the deviation of the magnetic field intensity that made the magnetic field more uniform.

Our previous study was established around the chitosan-collagen conduits. It was prepared following the procedures described previously (13). Briefly, the type I collagen (2.8 wt.%; Sigma, St. Louis, MO, USA) and chitosan (0.7 wt.%; Sigma, St. Louis, MO, USA) were mixed and dissolved in a solution of 0.1 Macetic acid (PH 3.2) at 4°C. After the mixture was centrifuged, the suspension was degassed and injected into a self-designed mold. After the mold was lyophilized for 48 h, the conduit was removed from the mold and cut into cylinders (12 mm in length, 1.5 mm in inner diameter, and 2.5 mm in outer diameter). Additionally, the conduits were cross-linked with a solution of genipin (1 wt.%, Challenge Bioproducts, Taichung, Taiwan) for 48 h, rinsed three times with distilled water, dehydrated for 30 min with 95% of ethanol, and air dried for 1 week. Before the surgery, the conduits were sterilized with an exposure to 20 kGy ^60^Co radiation (Figure 1A).

For microstructural observations, the conduits were washed three times with distilled water and then dehydrated in serial ethanol solutions followed by a brief vacuum drying. Thereafter, the dry samples were sputter-coated with gold at 40 mA, the microstructure of conduits was examined under a scanning electron microscope (S-3400N; HITACHI, Tokyo, Japan) at an accelerating voltage of 5 kV. The mean diameter of interconnected micropores on the wall was 21.37 ± 3.98 mm (range, 23.74–57.63 mm) (Figures 1B–D).

### Preparation of PEMF Stimulators and Determination of PEMF Parameters

The PEMF stimulator apparatus (GHY-III, Fourth Military Medical University (FMMU), Xi’an, China; China Patent no. ZL02224739.4) was provided by the cooperating organization of this study (39). It included in a PEMF generator and several identical Helmholtz coils with 800 mm coil diameters (Figure 1E). In brief, the coils were placed with interval of 304 mm in the same axis. Each coil was composed by enameled coated copper wire with 0.8 mm diameter. This ubiety of three coils reduced the deviation of the magnetic field intensity that made the magnetic field more uniform. The magnetic field value along the axial direction of the coil was expressed by:

$$B(X)=\frac{\mu_{0}NIR^{2}}{2}\{{\left[R^{2}+{\left(a+x\right)}^{2}\right]}^{-\frac{3}{2}}+{\left[R^{2}+{\left(a-x\right)}^{2}\right]}^{-\frac{3}{2}}+k{\left(R^{2}+X^{2}\right)}^{-\frac{3}{2}}\}$$

where μ_0_ is the permeability of vacuum, *I* is the current through the coils, *R* is the radius of the coils, *a* is the distance between the central coil and the outside coil, *x* is the abscissa relative to origin, *N* is the number of turns of the outside coil, and *k* × *N* is the number of turns of the middle coil. By setting the parameters *a* = 0.7601R and *k* = 0.5315, the second and fourth derivative of *B*(*X*) will become 0 at the position of origin and then the maximum uniformity of the magnetic field extent will be obtained. Therefore, we obtain the number of turns of the central coil, 266 turns, and the number of turns of the outside coils, 500 turns, and the distance between the central coil and the outside coil, approximately 304 mm. The measurement accuracy of the electromagnetic feld output was confirmed with a Gaussmeter (Model 455 DSP; Lake Shore Cryotronics). A small 2 Ω resistor was laid correctly in series with the Helmholtz coils, and the wave shape and frequency were visualized by an oscilloscope (6000 series; Agilent Technologies, USA). The PEMF waveform consisted of a pulsed burst (burst width 5 ms; pulse width, 0.2 ms; pulse wait, 0.02 ms; burst wait, 60 ms; pulse rise and fall times: 0.3 μs, 2.0 μs) repeated at 15 Hz.

Based on others’ experiences (17, 19) and our preliminary experiments, the coils were wired to a pulse generator, which produced an electromagnetic field signal with a frequency of 2 Hz, produced the magnetic field with amplitude of 0.3 mT and a pulse duration of 20 ms.

### Animals, Surgical Procedures, and Experimental Design

All protocols involving the use of animals followed the Guide of the Care and Use of Laboratory Animals (National Institutes of Health publication No. 85-23, revised 1985) and were approved by Institutional Ethical Committee of the Forth Military Medical University.

One hundred ninety-eight male adult Sprague-Dawley rats, weighing approximately 200–220 g, were obtained from Laboratory Animal Center of FMMU and were randomly divided into three groups as shown in Table 1. The animals were housed with controlled temperature (24 ± 2°C), humidity (50–60%), and a 12:12 h light–dark cycle. The rats were acclimatized to the laboratory and habituated to the observation chamber for at least 30 min each day for 7 days before testing. During the surgery, all animals were anesthetized with 1% sodium pentobarbital solution (40 mg/kg, i.p.). Under aseptic conditions, the left sciatic nerve was exposed using a muscle-splitting incision. A segment of sciatic nerve was removed, leaving a15-mm-long defect after retraction of the nerve ends, and then sutured by three perineural 11/0 nylon. The nerve defect was bridged with the nerve conduit sutured to both the proximal and distal nerve stumps. In all animals, the skin was closed with 7/0 stitches. After surgery, all animals were returned to their cages and fed with food and water as libitum as usual.

**Table 1:** **Number of rats per group and time point allocated to different assessments**.

	CF group	DPEMF group	NPEMF group
**1 week**			
Expression of regeneration-related genes	6 (NO.13/56/89/103/134/168)	6 (NO. 22/46/81/119/141/178)	6 (NO.31/96/100/105/153/183)

**3 weeks**			
Expression of regeneration-related genes	6 (NO.2/16/37/48/101/121)	6 (NO.1/27/65/71/151/189)	6 (NO.11/69/72/135/162/165)

**4 weeks**			
Axonal regeneration and functional recovery assessment^a^	6 (NO.18/61/67/91/98/193)	6 (NO.9/33/49/145/157/198)	6 (NO. 38/44/77/92/139/171)
Fluoro-Gold (FG) retrograde tracing assessment	6 (NO.20/35/60/83/109/146)	6 (NO.25/51/78/104/140/192)	6 (NO.4/58/84/123/158/170)
Immunohistochemistry assessment	6 (NO.23/59/107/147/152/166)	6 (NO.5/17/32/80/111/142)	6 (NO.15/76/99/155/156/187)

**8 weeks**			
Axonal regeneration and functional recovery assessment^a^	6 (NO.8/10/86/125/143/154)	6 (NO.19/41/95/113/149/160)	6 (NO.7/66/85/88/126/150)
Immunohistochemistry assessment	6 (NO.30/52/128/130/137/174)	6 (NO.28/45/116/132/161/185)	6 (NO.12/39/55/57/114/177)
FG retrograde tracing assessment	6 (NO.26/117/159/164/167/195)	6 (NO.14/68/106/131/148/163)	6 (NO.34/54/90/108/120/122)

**12 weeks**			
Axonal regeneration and functional recovery assessment^a^	6 (NO. 6/29/63/115/118/136)	6 (NO. 21/73/94/112/124/127)	6 (NO.24/43/97/129/172/173)
FG retrograde tracing assessment	6 (NO. 36/47/53/87/93/110)	6 (NO. 40/79/82/181/182/191)	6 (NO. 50/64/70/133/138/197)
Immunohistochemistry assessment	6 (NO. 42/74/75/169/176/196)	6 (NO.62/102/144/175/188/190)	6 (NO.3/179/180/184/186/194)
Total number	66	66	66

*^a^Axonal regeneration and functional recovery assessment containing morphometric analysis of sciatic nerve, behavioral analysis, electrophysiological assessment, and histological analysis of target muscle*.

The rats in experimental groups were subjected to daily PEMF exposure from the second day after surgery. In details, in DPEMF group, rats were exposed to the PEMF 4 h during 7:00–11:00; in NPEMF group, rats were exposed to the PEMF 4 h during 19:00–23:00; in CF group, rats were normally fed up without PEMF exposure.

### Behavioral Analysis

Functional recovery was assessed by the walking track analysis and the sciatic functional index (SFI) was calculated. SFI were calculated at 4, 8, and 12 weeks after surgery (40). In brief, the rats were trained to walk across a narrow wooden track (1 m long and 7 cm wide) leading to a darkened box containing their familiar housing mates before surgery. Postoperatively, the rat’s hind paws were dipped in red dye (non-toxic) before walking in the track and the recordings continued until five measurable footprints were collected. Then the footprints were scanned, and the SFI were measured and calculated using Photoshop version 6.0 (Adobe Systems, Ottawa, ON, Canada) with the following formula:

$$\begin{array}{ll} \text{SFI}= & \left[-38.3\times (\text{EPL}-\text{NPL})/\text{NPL}]+[109.5\times (\text{ETS}-\text{NTS})/\text{NTS}\right]\\ & +[13.3\times (\text{EIT}-\text{NIT})/\text{NIT}]-8.8 \end{array}$$

where print length (PL) is the distance from the heel to the top of the third toe; intermediary toe (IT) spread is the distance from the second to the fourth toe; and toe spread (TS) is the distance between the first and the fifth toe. The NPL, NTS, and NIT represent the PL, IT, and TS recorded from the non-operated foot, respectively; EPL, ETS, and EIT represent the PL, IT, and TS recorded from the operated, experimental foot, respectively. An SFI value that oscillates around 0 indicates better recovery, whereas an SFI value around 100 represents total dysfunction.

After the walking track analysis, a plantar test was performed for evaluation of heat hypersensitivity and sensory functional recovery of the injured hindlimb. In brief, each animal was placed in a clear Plexiglas box and radiant heat was applied to the left hind paw. Time from initial activation until paw withdrawal to in response to the heat was recorded. The test was repeated in 15 min intervals. The heat stimulation would be stopped to prevent thermal injury if the animals do not withdraw the paw for 30 s.

### Electrophysiological Assessment

Electrophysiological measurements were performed on the experimental animals before they were scarified for histological analysis. All rats were anesthetized prior to electrophysiological studies at 4, 8, and 12 weeks after surgery. Repair site was identified and insulated from the surrounding muscle with a rubber dam. A bipolar stimulating electrode was placed under the sciatic nerve at a location 10 mm proximal to the graft site. A recording electrode was placed in the gastrocnemius muscle. Then, the compound muscle action potentials (CMAPs) were recorded with a Power Lab 4SP distal data acquisition system (Keypoint 3.02 Denmark). For quantitative analysis, the peak amplitude of CMAP, latency of CMAP onset and nerve conduction velocity (NCV) values were calculated, respectively (41).

### Fluoro-Gold (FG) Retrograde Tracing

After the electrophysiological tests, retrograde labeling was performed and back-labeled cells were counted. In brief, the sciatic nerve was exposed and the nerve at the injection point 5 mm distal to the distal end of the conduit was crushed using a pair of forceps three times for 10 s with an interval of 10 s to make regenerated axons injured and 5 ml of 4% FG (Biotium, Hayward, CA, USA) solution was intraneurally injected into nerve trunk at the point described above followed by suture of incision. The rats were then kept routinely in their cages for 7 days. After 1 week, the rats were intracardially perfused with 4% (w/v) paraformaldehyde in 0.1 M phosphate buffer under anesthesia. The lumbar spinal cord was exposed and the L4, L5, and L6 together were harvested with the dorsal root ganglia (DRG), then postfixed in buffered 4% paraformaldehyde for 4 h, cryoprotected in 30% sucrose overnight at 4°C, and then sectioned on a cryostat. 25-mm thick transverse sections for spinal cords and 16 mm thick longitudinal sections for DRG were mounted on glass slides, viewed and photographed under a fluorescent microscope (DM6000; Leica, Germany). The number of FG-labeled spinal cord motoneurons and the number of FG-labeled DRG sensory neurons were counted directly.

### Immunoflourescence Assay of Regenerated Nerve

At 4 weeks after surgery, serial longitudinal sections (thickness of 10.0 mm) of the middle portions of the regenerated nerve were cut on a cryostat as described above after the fixation in 1% paraformaldehyde and the dehydration in 3% sucrose solution and collected in phosphate-buffered saline (PBS) to be processed immunohistochemically as free-floating sections. In brief, specimen-containing slides were incubated with 0.2% Triton X-100 for 10 min and 0.1% BSA for 30 min in the room temperature first. Then, they are stained overnight by anti-S100 protein rabbit monoclonal antibody (1:200; S1318; Bioworld, USA) and anti-NF200 protein mousemonoclonal antibody (1:200; 2836S; CST, USA) in 4°C. Next, the primary antibodies were probed with goat anti-rabbit IgG TRITC secondary antibody (1:200; ab150080; Abcam Inc., UK) and goat anti-mouse IgG FITC (1:200; ab150113; Abcam Inc., UK) for 2 h at 37°C. Then, the slides were incubated with DAPI (1:500 in PBS) at room temperature for 15 min. The specimens were rinsed three times (for 10 min) in PBS (pH 7.4) between each step. For double immunofluorescence assay, four slides were randomly collected in each group and each section was rinsed, mounted on glycerin coated slides, and cover slipped. Sections were analyzed by fluorescence microscopy (DM6000; Leica, Germany).

### Morphometric Analysis of Axonal Regeneration

At 4, 8, and 12 weeks after surgery, the regenerated nerves that formed in the place of the grafts were quickly harvested and fixed in 3 wt.% glutaraldehyde. After post-fixation in 1% osmium tetroxide in 0.1 M sodium cacodylate buffer (pH 7.3) for 2 h at 4°C, the tissues were dehydrated and embedded in epoxy resin embedding media. Then, the transverse semithin (thickness: 1.0 mm) and ultrathin sections (thickness: 50.0 nm) were cut from the distal portions of the regenerated nerve. The semithin sections were stained with a 1% toluidine blue/1% borax solution prepared in distilled water and examined under a light microscope (AH3; Olympus). Ultrathin sections were stained with uranyl acetate and lead citrate and were examined under a transmission electron microscope (H-600; HITACHI). For quantitative analysis, five semithin sections and five ultrathin sections were randomly selected at each portion of the regenerated nerve. The axonal regeneration was estimated by (1) the total area of regenerated nerves per semithin section, (2) the total number of myelinated axons per semithin section, and (3) the mean diameter of the nerve fibers per ultrathin section. The degree of myelination was estimated by the axon-to-fiber diameter ratio (*G*-ratio) per ultrathin section. Morphometric evaluations were completed by an investigator who was blinded to the study conditions.

### Histological Analysis of Target Muscles

Then, 12 weeks after surgery, the gastrocnemius muscles of operated hind limb were harvested and immersed in 4% paraformaldehyde in 0.1 mol/L phosphate buffer at 4°C for 2 weeks. Then five transverse sections (thickness of 50.0 mm) per specimen were prepared and subjected to HE staining. Thereafter, these sections were examined under a light microscope (AH3; Olympus). Five middle-powered fields (200) in each section were randomly chosen for quantitative analysis with a Leica software package. The extent of the atrophy/reinnervation of target muscles was assessed by the percentage of muscle fiber area (*P*_m_), which was calculated according to the equation:

$$P_{\text{m}}=A_{\text{m}}/A_{\text{t}}\times 100\%$$

where *A*_m_ is the area of muscle fibers in each field (magnification, 100) and *A*_t_ is the total area including muscle fibers and other tissues of the field.

### Real-time PCR (RT-PCR) Assay of Vascularization and Regeneration-Related Genes Activation

Also, 1 and 3 weeks after surgery, the regenerated nerves that formed inside the conduits were harvested, powdered in a glass homogenizer under liquid nitrogen, and lysed with lysis buffer (Promega, Madison, WI, USA). Total RNA was extracted and purified using an RNeasy column (Qiagen, Valencia, CA, USA). Then, RT-PCR was performed according to the manufacturer’s instructions. The sequencers of primers for nerve growth factor (NGF), brain-derived neurotrophic factor (BDNF), S-100, and β-actin (internal control) are shown in Table 2. The PCR reaction was conducted using 25 μl of sample cDNA, 2.5 μl of 10 PCR buffer, 2.0 μl of MgSO_4_ (25 mM), 2.5 μl dNTP mix (2 mM), 0.5 μlTaq DNA Polymerase (2 U/μl), and 15.8 μl deionized H_2_O. The reaction mixture was heated to 95°C or 2.5 min and then amplified for 40 cycles as follows: 95°C for 35 s (denaturation), 54°C for 30 s (annealing), and 65°C for 5 s (extension).

**Table 2:** **Primer sequences used for the real-time PCR**.

Target gene	GenBank accession no.	Direction	Sequence	*T*_m_
β-Actin	NM_031144.2	Upper	5′ ATGAAGATCCTGACCGA 3′	*F* = 60.29
		Lower	5′ GCTCATTGCCGATAGTG 3′	*R* = 60.97
Nerve growth factor	XM_227525.6	Upper	5′ TTTTGCCTTTGCCTGGT 3′	*F* = 62.35
		Lower	5′ GTTGATTGGCTGTGTCC 3′	*R* = 61.97
Brain-derived neurotrophic factor	NM_012513.4	Upper	5′ GCCCAACGAAGAAAACC 3′	*F* = 61.18
		Lower	5′ CCAGCAGAAAGAGCAGA 3′	*R* = 61.73
S100b	NM_013191.1	Upper	5′ ATCAGGTGCTCTCTTGAA 3′	*F* = 61.44
		Lower	5′ GTAACAGTGAAGCGACC 3′	*R* = 61.83

### Statistical Analysis

All data were expressed as mean SEM. The data were analyzed using one-way analysis of variance with the SPSS 13.0 software package (SPSS Inc., Chicago, IL, USA). Bonferroni test for pairwise comparisons was used to examine the effects of time and treatments. Values of *p* < 0.05 were considered statistically significant.

## Results

### Effect of Chronotherapy with PEMF on Axonal Regeneration

**Figure 2:**
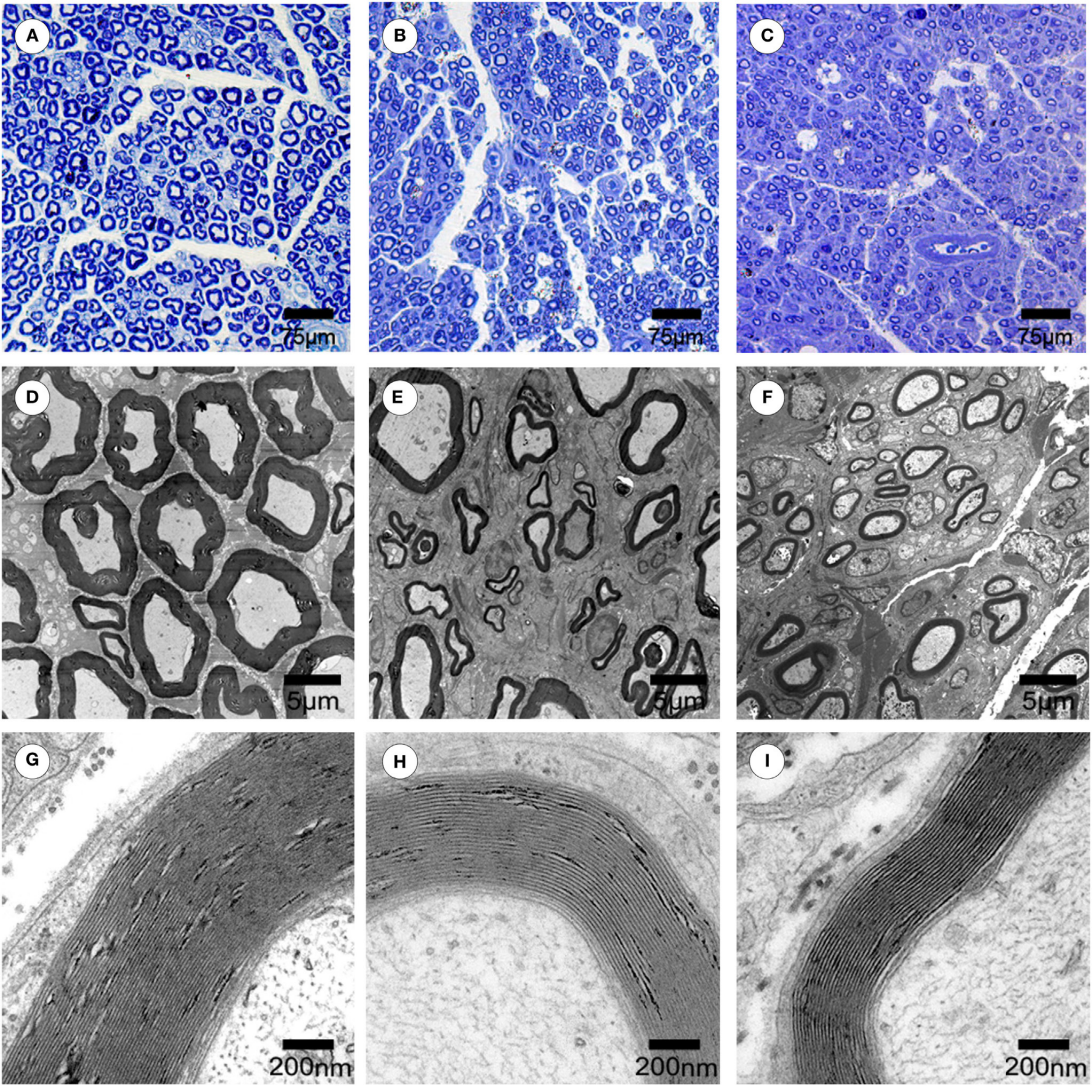
**Morphological appearance of regenerated nerves in each group**. The representative toluidine blue staining of regenerated axons **(A–C)** in the midst of conduit in the DPEMF group **(A)**, NPEMF group **(B)**, and CF group **(C)** at 12 weeks after surgery, respectively. The representative electron micrographs of regenerated axons **(D–F)** and myelin sheath **(G–I)** in the midst of conduit in the DPEMF group **(D,G)**, NPEMF group **(E,H)**, and CF group **(F,I)** at 12 weeks postoperatively.

**Figure 3:**
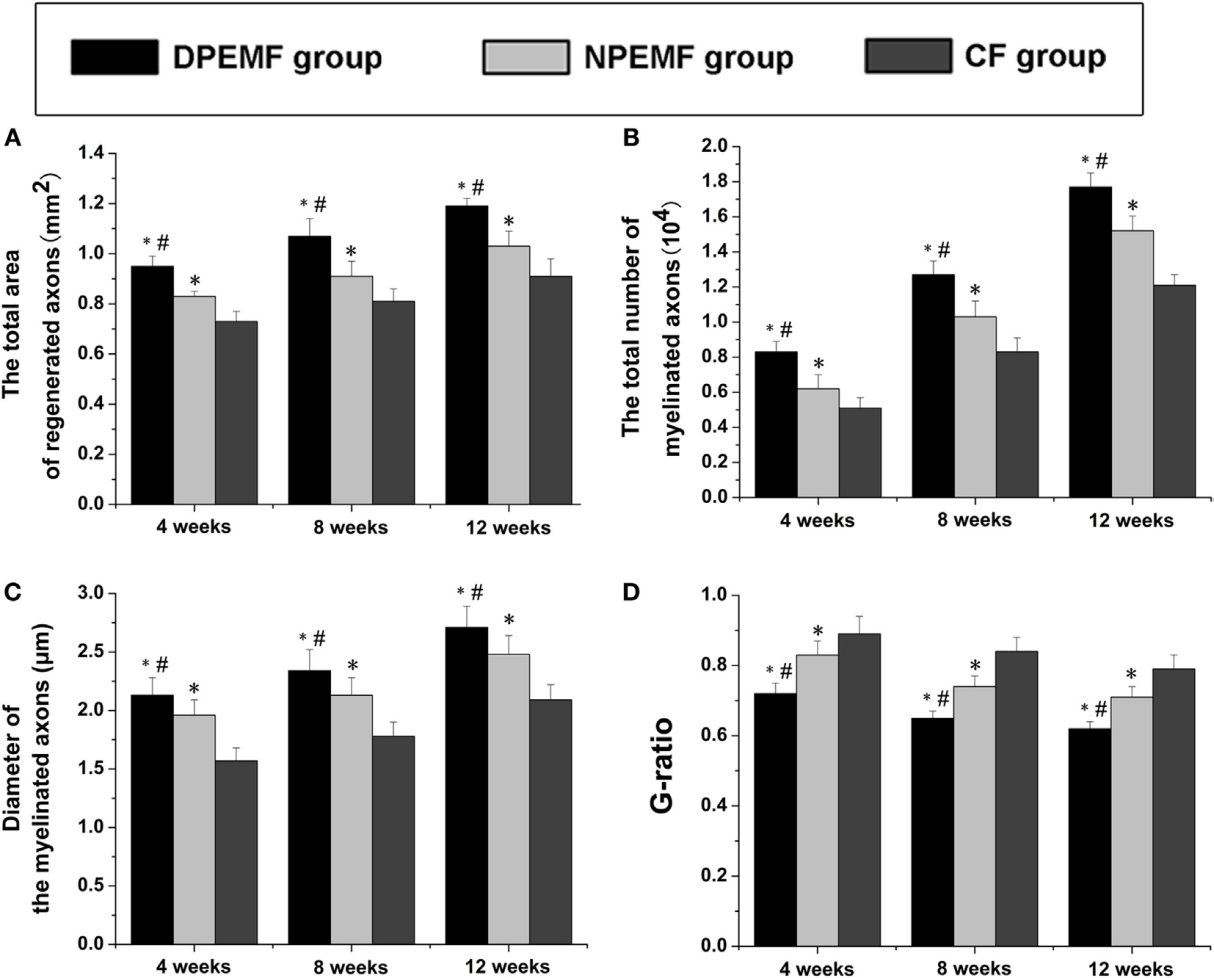
**Morphometric assessments of regenerated nerves in each group**. The cross-sectional area of regenerated nerve **(A)**, quantification of the myelinated axons **(B)**, the diameter of myelinated axons **(C)**, and *G*-ratios **(D)** in the midst portion of conduit **(A–D)**. All data were expressed as the mean ± SEM. *p < 0.05 for comparison with CF group, ^#^*p* < 0.05 for comparison with NPEMF group.

**Figure 4:**
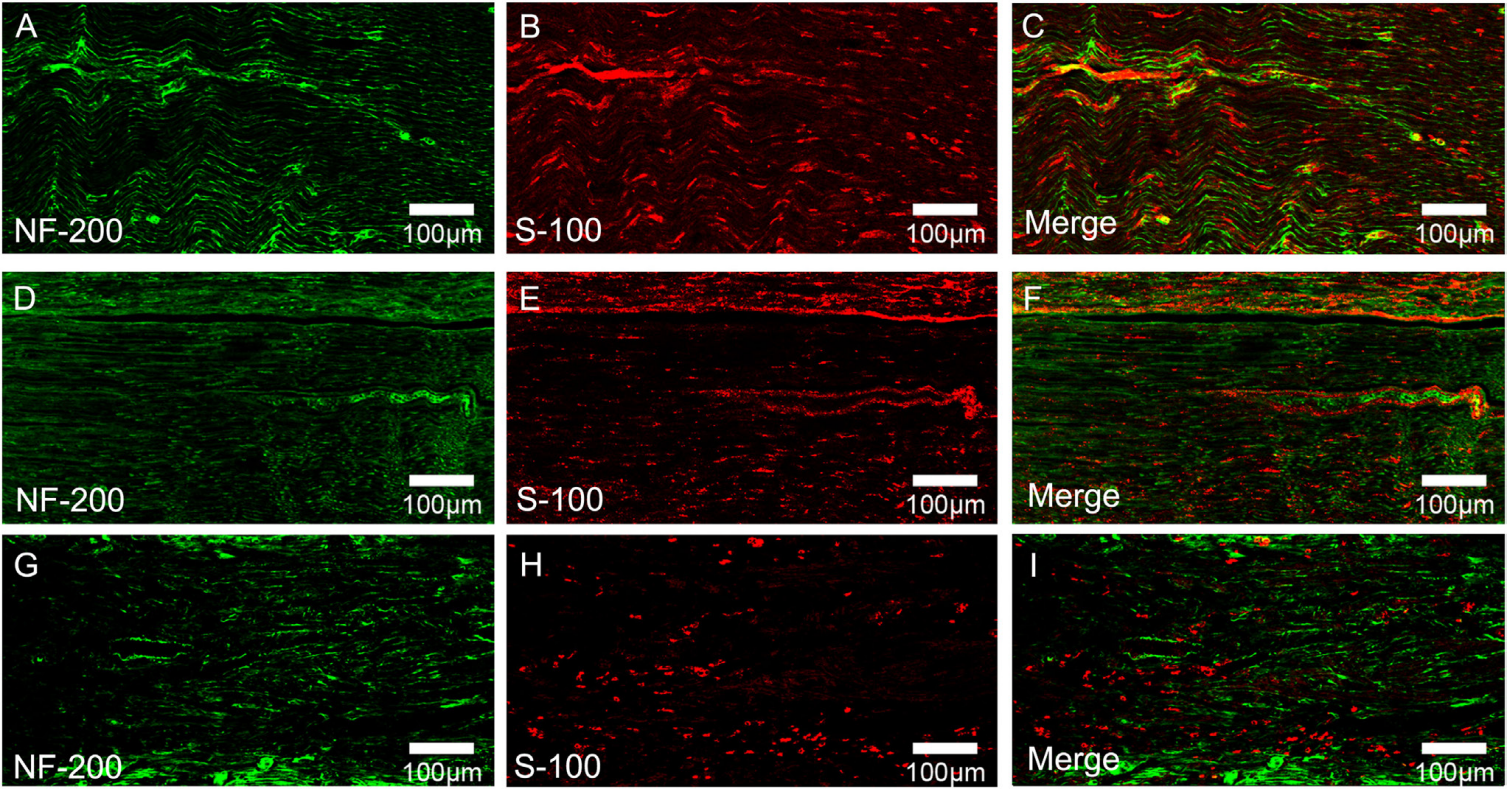
**Double-immunohistochemical staining for S-100 and NF200 in each group**. The representative images of regenerated nerves in the midst of conduit in the DPEMF group **(A–C)**, NPEMF group **(D–F)**, and CF group **(G–I)**, postoperatively.

To evaluate the effects of chronotherapy with PEMF on axonal regeneration, we examined the formation of regenerated axon using toluidine blue staining. We found that axonal regeneration was observed at the distal end at 12 weeks post implantation in all groups (Figures 2A–C). In the NPEMF group, the morphological appearance of regenerated axons was inferior to that in the DPEMF group (Figures 2D,E,G,H), but superior to that in the CF group (Figures 2F,I). In addition, the total area of regenerated axons (Figure 3A), the total number of myelinated axons (Figure 3B), and the mean diameter of the myelinated axons (Figure 3C) in the DPEMF and NPEMF groups were significantly higher than those in the CF group (Figure 3; *n* = 6, *p* < 0.05). Compared to NPEMF group, the parameters described above were much greater in DPEMF group. Further analysis showed that the degree of myelination (*G*-ratio) was much better in DPEMF group than that in the NPEMF and CF groups (Figure 3D; *n* = 6, *p* < 0.05). Furthermore, double S100/NF200 immunofluorescence analysis in the middle portions of the regenerated nerve showed that the migrated SCs and regenerated axons were even distributed in DPEMF group (Figures 4A–C), which showed better morphological appearance than those in the NPEMF and CF groups (Figures 4D–I).

### Effect of Chronotherapy with PEMF on Neurologic Function Recovery

**Figure 5:**
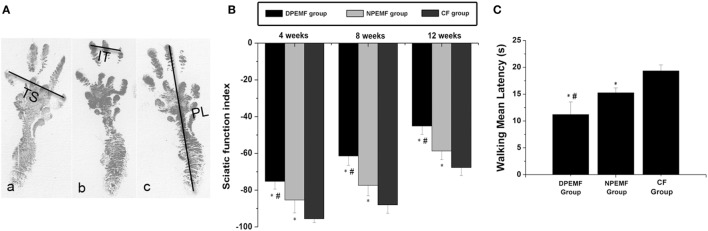
**The Sciatic function index and walking mean latency in each group**. The operative left footprints [**(A)** (a–c)] in the DPEMF group [**(A)** (a)], NPEMF group [**(A)** (b)], and CF group [**(A)** (c)] at 12 weeks postoperatively. All data were expressed as the mean ± SEM **(B,C)**. *p < 0.05 for comparison with CF group, ^#^*p* < 0.05 for comparison with NPEMF group.

**Figure 6:**
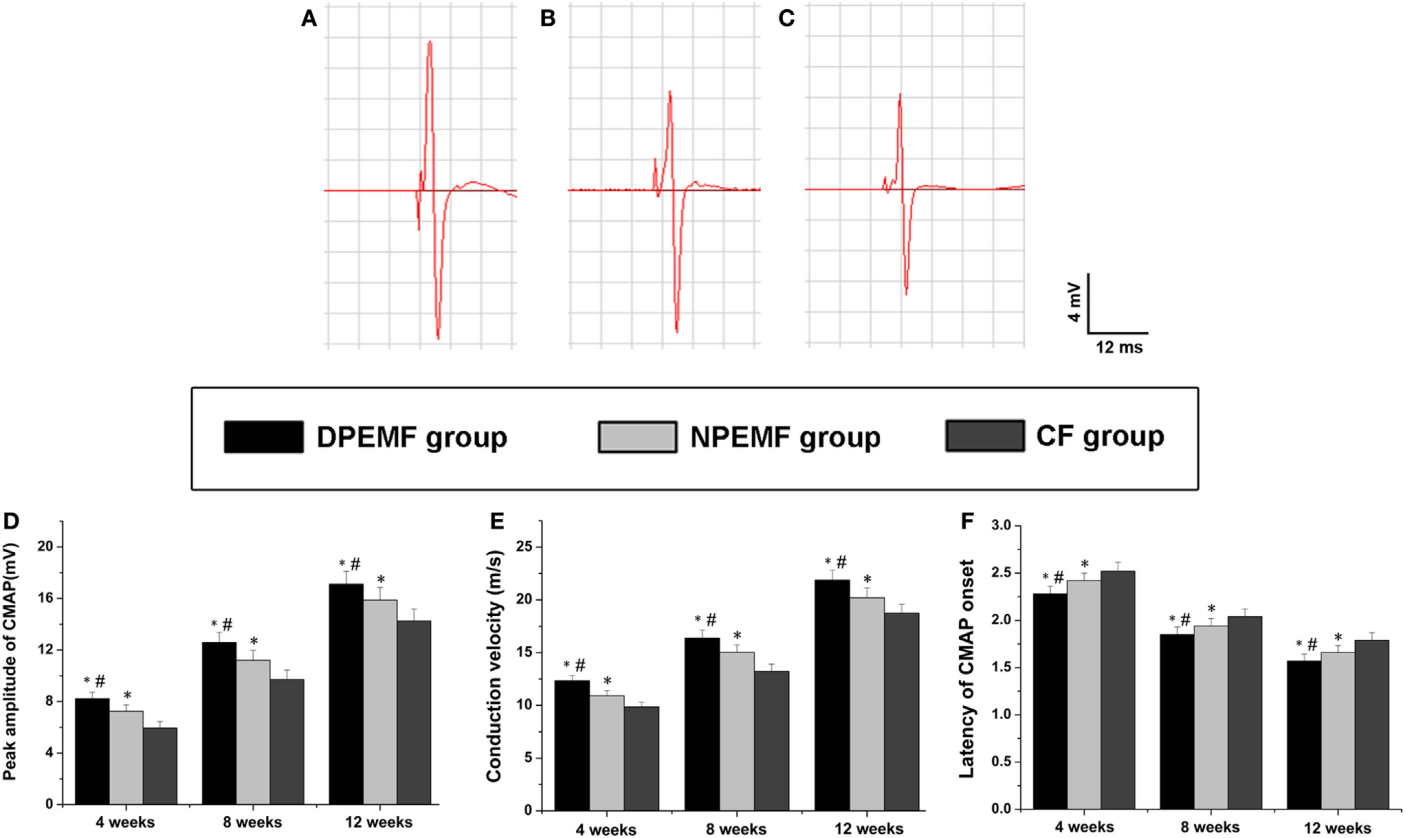
**The electrophysiological assessments in each group**. The representative recordings are shown from the DPEMF group **(A)**, the NPEMF group **(B)**, and the CF group **(C)** at 12 weeks postoperatively. The peak amplitude of compound muscle action potentials (CMAPs) **(D)**, NCV value **(E)**, and latency of CMAP onset **(F)** recorded at 4, 8, and 12 weeks after surgery. All data were expressed as the mean ± SEM. *p < 0.05 for comparison with CF group, ^#^*p* < 0.05 for comparison with NPEMF group.

Statistical analysis of the data collected from SFI measurement and electrophysiological assessment revealed that functional recovery was achieved in all groups at 4, 8, and 12 weeks after surgery. However, the values of SFI and the amplitude of CMAP and NCV were higher, but the latency of CMAP onset were shorter in DPEMF and NPEMF group than the ones in the CF groups (Figures 5A,B and 6A–F; *n* = 6, *p* < 0.05). In addition, the SFI values, amplitude of CMAP and NCV were significantly higher, and the latency of CMAP onset was significantly lower in the DPEMF group than the ones in the NPEMF group, indicating that better functional recovery was achieved in the DPEMF group in rats (Figures 5A,B and 6A–F; *n* = 6, *p* < 0.05). Sensory functional recovery was achieved in all groups at 12 weeks after surgery based on the plantar test. DPEMF group and NPEMF group showed a quicker response to thermal stimulus than the CF group. Furthermore, DPEMF group showed better sensory functional recovery compared with that NPEMF group (Figure 5C; *n* = 6, *p* < 0.05).

**Figure 7:**
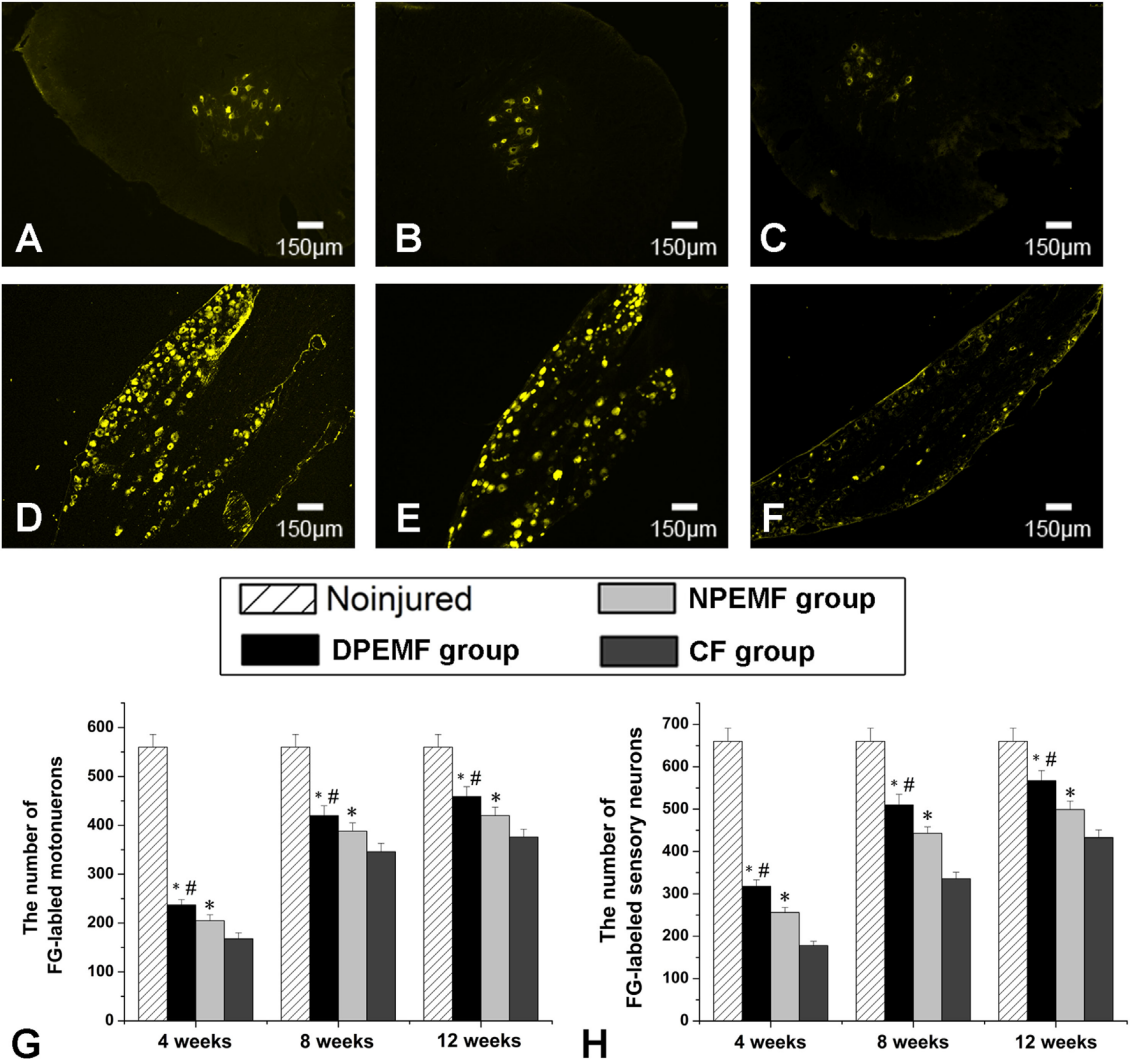
**Fluoro-Gold (FG) retrograde tracing in each group**. FG-labeled motoneurons in spinal cord **(A–C)** and sensory neurons in dorsal root ganglia **(D–F)** in the DPEMF group **(A,D)**, NPEMF group **(B,E)**, and CF group **(C,F)** at 12 weeks after surgery. The average number of FG-positive motoneurons and sensory neurons in each group were shown in panels **(G)** and **(H)**, respectively. All data were expressed as the mean ± SEM. *p < 0.05 for comparison with CF group, ^#^*p* < 0.05 for comparison with CFO group.

**Figure 8:**
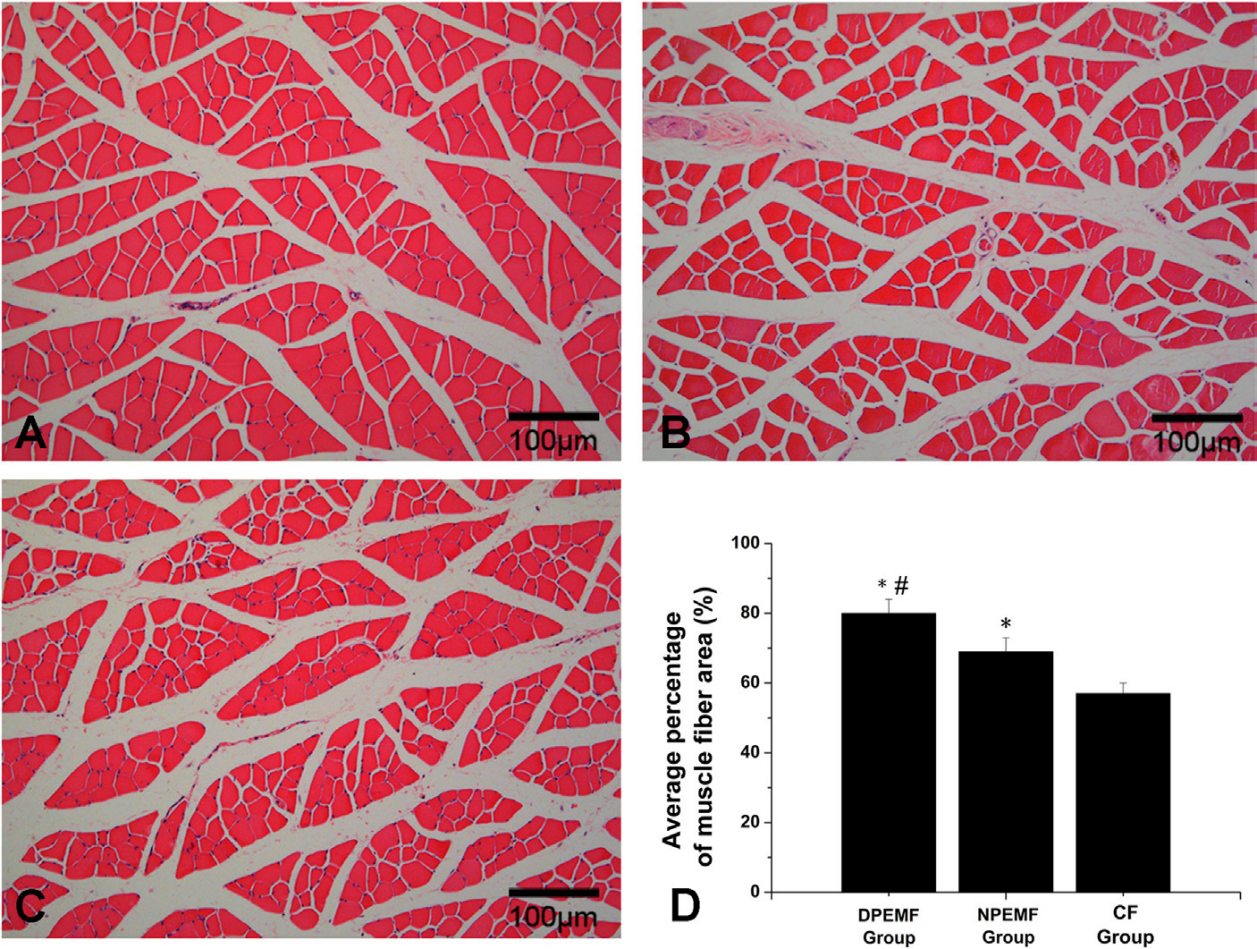
**Histological analysis of target gastrocnemius muscle in each group**. Representative light micrographs of the transverse-sectioned gastrocnemius muscle following HE staining for the operated limb in the DPEMF group **(A)**, NPEMF group **(B)**, and CF group **(C)** at 12 weeks postoperatively. The average percentage of muscle fiber in each group was shown in panel **(D)**. All data were expressed as the mean ± SEM. *p < 0.05 for comparison with CF group, ^#^*p* < 0.05 for comparison with CFO group.

The FG-positive cells were observed within both the anterior horn of spinal cord and DRG in all groups at the predefined time points. The number of FG-labeled motoneurons and sensory neurons in the DPEMF and NPEMF groups were significantly higher than those in the CF group (Figures 7C,F–H; *n* = 6, *p* < 0.05). In addition, the number of FG-labeled motoneurons and sensory neurons in the DPEMF group was significantly higher than that in the NPEMF group (Figures 7A,B,D,E,G,H; *n* = 6, *p* < 0.05). To evaluate the effect of chronotherapy with PEMF on motor functional recovery, the morphological analysis of gastrocnemius muscles was applied. As shown in Figures 8A–C, the percentage of gastrocnemius muscle fiber area (*P*_m_) in the DPEMF group and NPEMF group were significantly higher than that in the CF group. This finding indicates that the exposure to PEMF can effectively prevent muscle atrophy. In addition, *P*_m_ in the DPEMF group was significantly higher than that in the NPEMF group (Figures 8A,B,D; *n* = 6, *p* < 0.05).

### Effect of Chronotherapy with PEMF on Expression of Regeneration-Related Genes

**Figure 9:**
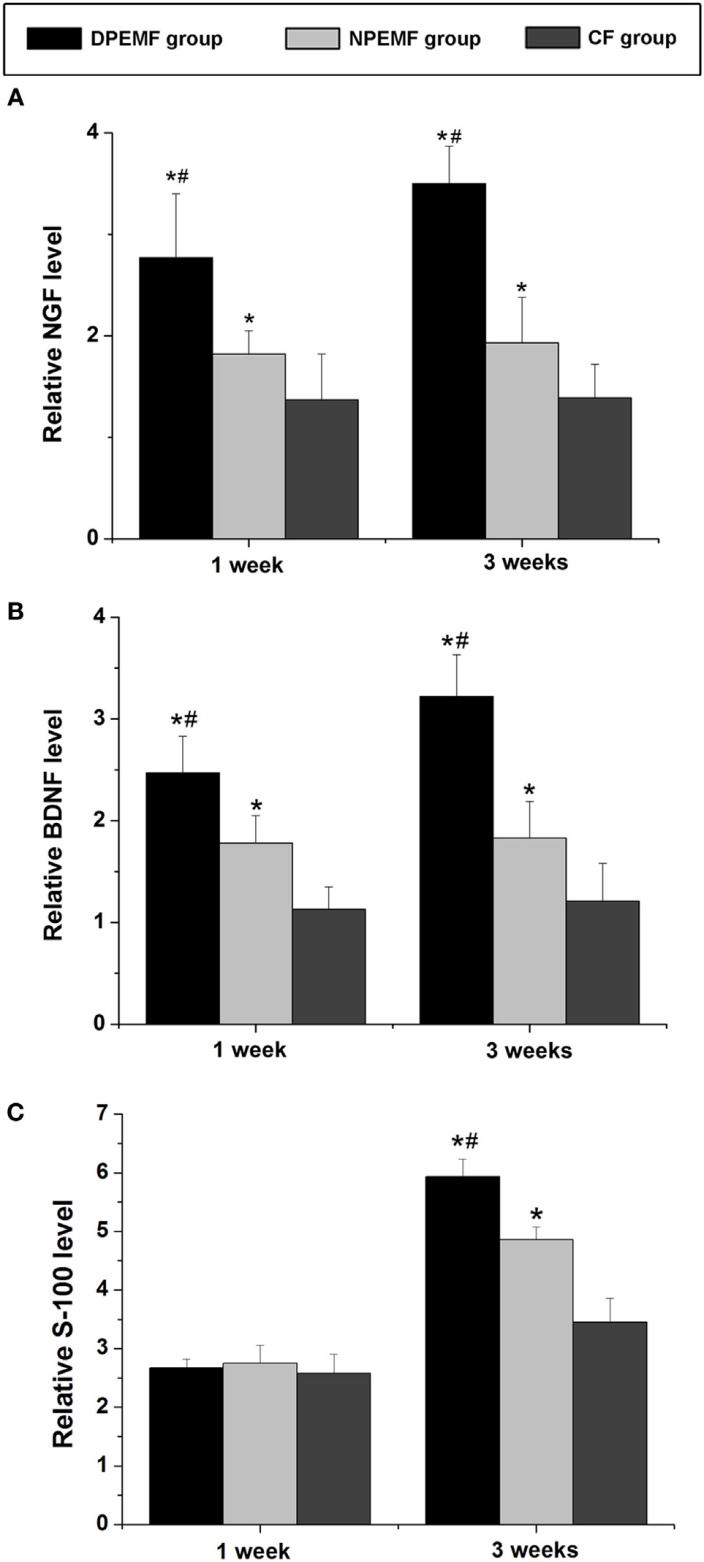
**The mRNA levels of brain-derived neurotrophic factor (BDNF), nerve growth factor (NGF), VEGF, and S-100**. The mRNA levels of **(A)** NGF, **(B)** BDNF, and **(C)** S-100 are determined for the DPEMF group, NPEMF group, and CF group at 1 and 3 weeks after surgery. Each test was repeated three times. All data were expressed as the mean ± SEM. *p < 0.05 for comparison with CF group, ^#^*p* < 0.05 for comparison with CFO group.

The mRNA levels of NGF, BDNF, and S-100 were examined by RT-PCR at 1 and 3 weeks after surgery. As shown in Figure 9, at 1 week after surgery, the mRNA levels of NGF in DPEMF group were 1.52-fold and 2.02-fold higher compared with those in NPEMF group and CF group, respectively (Figure 9A; *n* = 6, *p* < 0.05). The mRNA levels of BDNF in DPEMF group were 1.38-fold and 2.19-fold higher compared with those in NPEMF group and CF group, respectively (Figure 9B; *n* = 6, *p* < 0.05). However, the mRNA levels of S-100 were in the similar range among DPEMF group and NPEMF group and CF group (Figure 9C; *n* = 6, *p* > 0.05). At 3 weeks after surgery, the mRNA levels of NGF in DPEMF group were 1.81-fold and 2.52-fold higher than those in NPEMF group and CF group, respectively (Figure 9A; *n* = 6, *p* < 0.05). The mRNA levels of BDNF in DPEMF group were 1.76-fold and 2.66-fold higher compared with those in NPEMF group and CF group, respectively (Figure 9B; *n* = 6, *p* < 0.05). The mRNA levels of S-100 in the DPEMF group were 1.22-fold and 1.72-fold higher than those in NPEMF group and CF group, respectively (Figure 9C; *n* = 6, *p* < 0.05).

## Discussion

In this study, we investigated the effectiveness of chronotherapy with PEMF for improvement of axonal regeneration in bridging a 15-mm nerve defect using the chitosan-collagen conduit after peripheral nerve injury. Our *in vivo* study showed that the nerve regeneration in PEMF groups (both DPEMF group and NPEMF group) was significantly better than CF group, which indicates that PEMF stimulus is capable of promoting the peripheral nerve regeneration and functional recovery after injury. Moreover, CR plays an important role in treating peripheral nerve defects by PEMF stimulus. The results of histormorphometry and immunohistochemical analysis showed better morphological appearances in DPEMF group than those in NPEMF group. Further evaluation of motor functional recovery also revealed that the rats in DPEMF group achieved better curative effects than those in NPEMF group. In addition, the upregulation of NGF, BDNF, and S-100 mRNA levels at 3 weeks after surgery further proves the critical role of CR in the treatment of peripheral nerve regeneration by PEMF stimulus. Taken together, these findings indicate that CR dramatically influences the microenvironment of nerve regeneration, and the application of PEMF can optimize the electromagnetism microenvironment, hence improve the peripheral nerve regeneration and functional recovery. In addition, during the preliminary experiment of this study, we set daytime sham-radiation group and nighttime sham-radiation group as sham control groups, and we found that the results of these two groups have no significant differences compared with those in CF group. To avoid the increase of sample size and to be in compliance with Institutional Ethical Committee, CF group was served as the control group in the present study.

Optimizing the microenvironment of nerve regeneration after injury is always a hotspot in tissue engineering. At present, various nerve scaffolds were developed and showed beneficial effects on improving the nerve regeneration. An ideal material of nerve scaffolds should be non-toxic, porosity, and degradable and enough strength to provide microenvironment for migration of SCs and axon regeneration. Our collagen-chitosan conduits are fabricated based on our preliminary study (13). This conduit was developed with longitudinally oriented microchannels and a honeycomb-like inner framework, which are able to guide migration of SCs and axon extension. Furthermore, we optimized the techniques of nerve conduits. The magnetic nanocomposites were introduced into the existing collagen-chitosan materials, which makes the conduits with a good electromagnetical conductivity (42).

It has been proved that application of PEMF improves the curative effect of a wide range of orthopedic disorders including nerve injury, osteoarthritis, osteoporosis, etc. In recent years, it has been shown that the electromagnetic radiation by PEMF has both positive and negative biological effects (43). For the negative effects, it has been found that electromagnetic fields can elicit a series of stress responses, which causes the adaptive changes in rats’ hippocampus (44). In addition, electromagnetic fields also trigger to some extent the alteration of cellular morphology and function (20). However, it has not been studied, so far, if the types, parameters, and radiation methods of PEMF could be optimized to achieve beneficial effects, and if PEMF and CR have synergistically positive effects on nerve regeneration.

To systemically evaluate the synergistic effects of PEMF and CR on the treatment of peripheral nerve defects, we analyzed the effect of chronotherapy on axonal regeneration and neurologic function recovery. It is known that that morphometric indices play as a key role in measuring the quality of the regenerated nerves (45). Through the analysis of nerve histormorphometry, better morphological appearances, including the total area of regenerated nerves, the total number of myelinated axons, the mean diameter of the nerve fibers, and the axon-to-fiber diameter ratio (*G*-ratio), were found in the group with PEMF radiation compared to the group, which was simply bridged the gap by nerve conduits without PEMF. Moreover, those parameters described above in DPEMF group were significantly better than those in NPEMF group. We further estimated the effect of chronotherapy with PEMF on neurologic function recovery. Based on the observations, we found that the amplitude of CMAP, SFI values, the histological appearance of gastrocnemius muscles, as well as the number of FG-labeled neurons, were significantly higher in PEMF group than those in CF group. Although the improvement of neurologic function recovery was proven in both two PEMF groups, these parameters described above in DPEMF group were even higher than those in NPEMF group. These results indicate that more axons successfully regenerate most likely through the conduits into the distal stumps and reinnervate target muscles under the effect of PEMF. These findings suggest that PEMF provide a favorable electromagnetism microenvironment during the regrowth process of injured nerves not only to improve the regeneration quality but also to control the regeneration orientation. The outcomes in PEMF groups are likely attributed to the direct beneficial effects of PEMF radiation. Moreover, the daytime PEMF radiation provides better curative effects in rats, including axonal regeneration and functional recovery.

It is known that SCs are the most essential cells in peripheral nerve regeneration. As the leading glia cells in peripheral nervous system, its proliferation and migration orientation guide the regenerated axons (46). In addition, SCs release many neurotrophic factors, such as NGF and BDNF, during the regeneration process (47, 48). In present study, we found that PEMF upregulated the mRNA levels of S-100 at the local site of nerve defects at 3 weeks after surgery. However, no difference was observed among all groups at 1 week after surgery. The results suggest that PEMF can improve the proliferation of SCs, but not at the early period (1 week) after injury. Moreover, the mRNA levels of NGF and BDNF were also upregulated by PEMF at 1 and 3 weeks after surgery, indicating that PEMF treatment helps to establish a preferred microenvironment after nerve injure. It may be also attributed to the beneficial effect of PEMF on endogenous SCs. Interestingly, the expression of regeneration-related genes were also affected by CR. The mRNA levels of those genes in DPEMF group were significantly higher than those in NPEMF except for the mRNA levels of S-100 at 1 week after surgery. These findings suggest that CR play an important role in the beneficial effect of PEMF on the proliferation and vitality of SCs.

The mechanism of chronotherapy in PEMF treatments of nerve regeneration is still unknown. The better curative effect achieved by PEMF radiation in daytime may probably be attributed to the vitality of cells and activity of neuroendocrine system. It is known that CR, as well as hormones, enzyme, plays an essential role in the daily behaviors of all species, influencing the neural activity, metabolism and motor function, etc. (49–51). As the most fundamental components of creatures, cells and their physiological functions are also influenced by CR in rat model (52, 53). Those results suggest that the daily activity and proliferation of cells are influenced by the CR, and the cellular activity is more dynamic during the daytime than the one during the nighttime in rodent rats. In the present study, the better effectiveness of PEMF radiation in daytime on nerve regeneration might be due to the higher cellular activity during daytime. On the other hand, neuroendocrine system might be affected by CR indirectly. In recent years, melatonin, which is secreted by conarium, has been increasingly recognized to improve nerve regeneration and functional reestablishment after nerve injury (54, 55). Additionally, the dose of melatonin must be high enough to achieve a beneficial effect on nerve regeneration. It has been shown that the levels of melatonin have a circadian fluctuation during the dark–light cycle (56), and there is close relationship between melatonin and CR during the melatonin treatment on nerve injury (57). In addition, other hormones, such as growth hormone (GH) and the related insulin growth factor-1, have also been shown to enhance the survival of motor fibers and axonal branching (58–61). It is interesting to further study how CR modulates the effect of PEMF on secretion of those hormones and the role of this effect in the process of peripheral nerve regeneration.

Pulsed electromagnetic fields radiation can alter the endogenous circadian clocks in animals by affecting their sleep–wake cycles (62). However, it is still unclear whether this influence differs with the radiation time and how it works during peripheral nerve regeneration. Furthermore, the mechanisms underlying the role of CR in the beneficial effect of PEMF on nerve regeneration remain elusive. In addition, we hope to apply our findings in clinical trials. At present, the nerve conduits have been widely used in clinic and showed beneficial effects on improving the peripheral nerve regeneration after injury (63, 64). The concept of CR and PEMF effects derived from our study may further optimize the present therapy methods by using nerve conduits clinically. It may provide individual rehabilitation therapy scheme. For example, patients may achieve PEMF radiation at home using the portable electromagnetic therapeutic apparatus instead of going to health recovery center for rehabilitation.

In summary, daytime PEMF radiation is more efficient in promoting nerve regeneration than nighttime radiation in rats. However, considering that rats and humans show opposite sleep–wake cycles and circadian physiological activities (25), which present approximately 12 h offset in humans compared to those in rats, we speculated that, in humans, PEMF radiation in nighttime may be more advantageous for axons regeneration compared with radiation in daytime. In addition, in humans, melatonin secretion in the nighttime is fivefold to tenfold higher than that in the daytime and GHs also present similar circadian fluctuations with a peak during nighttime (65, 66). Therefore, it is an important subject for further study to research how CR affects the beneficial effect of PEMF on nerve regeneration in humans.

## Author Contributions

ZLuo, JH, and SZ conceived the study and participated in its design and coordination. SZ, JG, YY, and LL performed experiments and acquired data. SZ, JG, and DJ performed the analysis. MR, MW, and LH interpreted the data. SZ and JH wrote the manuscript. All authors read and approved the final manuscript.

## Conflict of Interest Statement

The authors declare that the research was conducted in the absence of any commercial or financial relationships that could be construed as a potential conflict of interest.
